# Editorial: ADAM10 in Cancer Immunology and Autoimmunity: More Than a Simple Biochemical Scissor

**DOI:** 10.3389/fimmu.2020.01483

**Published:** 2020-07-16

**Authors:** Armando Rossello, Alexander Steinle, Alessandro Poggi, Maria R. Zocchi

**Affiliations:** ^1^ProInLab, Department of Pharmacy, University of Pisa, Pisa, Italy; ^2^Institute for Molecular Medicine, Goethe-University, Frankfurt am Main, Germany; ^3^Frankfurt Cancer Institute, Frankfurt am Main, Germany; ^4^Molecular Oncology and Angiogenesis Unit, IRCCS Policlinico San Martino, Genoa, Italy; ^5^Division of Immunology Transplants and Infectious Diseases, IRCCS San Raffaele Scientific Institute, Milan, Italy

**Keywords:** metzincin, NKG2D, NKG2DL, CD30, Hodgkin lymphoma, ADAM10 exocyte, platelets

Altered expression of the ADAM (A Disintegrin and Metalloproteinase) proteins, usually involved in biological processes such as proteolysis, cell adhesion, proliferation, migration, and signaling, has been associated with several diseases including asthma, arthritis, neurodegenerative diseases, atherosclerosis, and cancer (1–4). Also, ADAM10 is involved in the pathogenesis of autoimmune diseases such as multiple sclerosis or systemic lupus erythematosus, and the development of inflammation or allergy (5, 6). This Special Issue is focused on the pathophysiological role of ADAM10 in tumors and autoimmunity, including potential therapeutic targeting of this enzyme with specific inhibitors.

The best-characterized function of ADAM10 is the proteolytic cleavage of different transmembrane proteins, a process known as “ectodomain shedding” that targets the extracellular domain of several types of cell surface molecules (1, 2). Other functions of this enzyme are not directly related to the activation of its catalytic domain but rather due to its exosite, that is a secondary substrate-binding site (7).

In particular, ADAM10 has been reported to shed the “stress-induced” molecules MICA, MICB, and ULBPs expressed on the cancer cell surface (8–11). These molecules are responsible for inducing an immune response against cancer cells upon binding to NKG2D receptors that are expressed on natural killer (NK) cells and most cytotoxic T lymphocytes. The ADAM10-mediated proteolytic shedding of these NKG2D ligands (NKG2DL) into the extracellular milieu can impair the recognition of cancer cells by T or NK cells (9–11). This mechanism has been evidenced in many types of tumors including melanoma, various carcinomas, and hematopoietic malignancies such as chronic lymphocytic leukemia, acute myeloid leukemia, non-Hodgkin and Hodgkin's lymphomas (12, 13). In the latter neoplasia, ADAM10-mediated CD30 shedding is reported to impair the recognition of this molecule by therapeutic monoclonal antibodies, in addition to the reduced immune surveillance through enhanced NKG2DL shedding (12–14).

The contribution by Zingoni et al. provides a topical overview of the tumor-associated up-regulation of NKG2DL and the cell stress-regulated ADAM10 activity mediating NKG2DL shedding in the context of carcinogenesis and cancer therapy. They highlight enhanced NKG2DL shedding in response to chemotherapy-induced cellular senescence of tumor cells as a consequence of both, induced NKG2DL expression and ADAM10 activity. Similarly, therapeutic targeting of the DNA damage response (DDR) affects the release of soluble NKG2DL by tumor cells through induction of NKG2DL and modulating ADAM10 expression and activity. They emphasize that targeting ADAM-mediated shedding of NKG2DL in the course of cancer therapies may restore immune detection and elimination of tumor cells via the NKG2D axis.

Hansen et al. explain how CD30 processing, due to the activity of ADAM10, might influence the impact of CD30 antibody-drug conjugates, such as Brentuximab Vedotin, reducing their efficacy in Hodgkin lymphomas, as previously described by the same group. This review evidences that the enzyme is catalytically active in extracellular vesicles and gradually releases sCD30, that can be measured in the patients' plasma, creating a “crossfire effect” that may modulate the response to therapy (16).

In turn, Maurer et al. point out a peculiar function of platelet-associated ADAM10. ADAM10 is highly expressed by platelets, where it is not only of major relevance in regulating hemostasis but also appears to contribute to the metastasis-promoting effect of platelets. This review comprehensively lists ADAM10 target structures of platelets and discusses various modes of ADAM10-mediated shedding including canonical shedding (in cis) and non-canonical shedding (in trans). Further, the authors summarize new insights into the world of proteins involved in ADAM10 processing, trafficking, and modulation such as TspanC8 tetraspanins, as reported by others (15), and TIMPs. Overall, this review illustrates the multifaceted role of ADAM10 expressed by platelets.

For all these reasons, in the last decade, an increasing interest has emerged toward the development of selective ADAMs ligands for their potential use for early-stage diagnosis and therapy of cancer (16–19). Several ADAM10 inhibitors proved to be effective in reducing tumor cell growth, inducing anti-tumor immune reactions or enhancing the effect of therapeutic antibody-drug conjugates *in vitro*. Examples are given by studies in gliomas, solid cancers, and hematologic tumors, including Hodgkin lymphoma (14, 20–24).

Some recent ADAM10 blockers proved to rescue both anti-tumor effect of Brentuximab Vedotin and sensitivity of Reed-Sternberg cells to effector lymphocytes, in particular through the antibody-dependent cellular cytotoxicity elicited by the therapeutic monoclonal antibody Iratumumab (20–24). Interestingly, these inhibitors were also carried by exosomes, making them able to spread their effects into the microenvironment (24). This points to the importance of targeting ADAM10 on different cell types, since exosomes can be released, for instance, by mesenchymal stromal cells or fibroblasts or accessory cells at the site of the lesion (24, 25). Very recently, cleavage of PD-L1 from lymphoma and solid tumor cells by ADAM10 and ADAM17 has been reported (26, 27). The consequent release of soluble PD-L1 was shown to induce apoptosis of immunocompetent CD8 T cells leading to an impairment of the anti-tumor immune response (27). This mechanism may confer resistance to PD-(L)1 blockers, thereby playing a role in tumor-mediated immunosuppression. Hence, it is conceivable to consider ADAM10/17 inhibitors also for an improvement of immunotherapies targeting the PD-1/PD-L1 axis.

However, despite the considerable number of studies generating significant data, the clinical trials have not confirmed the initial encouraging results and effective compounds are still missing.

The contributions by Smith et al. and by Minond et al. face this problem from two different viewpoints. The former reports on recent pre-clinical data with inhibitors and clinical data supporting the use of ADAM10 inhibitors in cancer and autoimmunity, searching for a mean to improve the potency and efficiency of anti-ADAM10 products alone or paired with other drug treatments (Smith et al.). The latter introduces the importance of ADAM10 non-catalytic domain, called exosite, addressing the possibility to target the exosite and, in particular, the glycosylation sites of ADAM10 (Minond). This suggests that proteolysis of specific ADAM10 substrates involved in various diseases can be targeted using knowledge on their glycosylation as well as on differences in their non-catalytic domains (28, 29). These results may open new avenues to circumvent the poor selectivity of inhibitors for ADAM10 and/or for ADAM10 substrates that currently represents the main obstacle to develop efficient drugs. Such novel targeting concepts introduce a new perspective for therapeutic approaches involving ADAM10 inhibitors in a wide spectrum of diseases.

## Author Contributions

MZ, AR, AP, and AS planned, wrote, and revised the editorial manuscript. All authors contributed to the article and approved the submitted version.

## Conflict of Interest

The authors declare that the research was conducted in the absence of any commercial or financial relationships that could be construed as a potential conflict of interest.
